# RDASNet: Image Denoising via a Residual Dense Attention Similarity Network

**DOI:** 10.3390/s23031486

**Published:** 2023-01-29

**Authors:** Haowu Tao, Wenhua Guo, Rui Han, Qi Yang, Jiyuan Zhao

**Affiliations:** 1 School of Computer Science and Technology, Xi’an Jiaotong University, Xi’an 710049, China; 2 State Key Laboratory for Manufacturing Systems Engineering, Xi’an Jiaotong University, Xi’an 710049, China

**Keywords:** image denoising, CNN, attention similarity module, residual dense block

## Abstract

In recent years, thanks to the performance advantages of convolutional neural networks (CNNs), CNNs have been widely used in image denoising. However, most of the CNN-based image-denoising models cannot make full use of the redundancy of image data, which limits the expressiveness of the model. We propose a new image-denoising model that aims to extract the local features of the image through CNN and focus on the global information of the image through the attention similarity module (ASM), especially the global similarity details of the image. Furthermore, dilation convolution is used to enlarge the receptive field to better focus on the global features. Moreover, avg-pooling is used to smooth and suppress noise in the ASM to further improve model performance. In addition, through global residual learning, the effect is enhanced from shallow to deep layers. A large number of experiments show that our proposed model has a better image-denoising effect, including quantitative and visual results. It is more suitable for complex blind noise and real images.

## 1. Introduction

As an important information carrier, imaging is widely used in remote sensing, medicine, aerospace, and other fields. However, due to the interference of imaging equipment and external factors, images are very easily affected by various noises and become blurred [1], so image denoising is particularly important. In fact, image denoising has always been a fundamental and important problem in computer vision [2]. Its purpose is to recover a clean image from a noisy image [3]. In general, for noisy images *y*, the image-denoising problem can be expressed as y=x+v, where *x* is the original image and *v* represents additive Gaussian noise (AWGN) with a standard deviation of σ.

From the point of view of Bayes, when the possibility is known, imaging prior modeling is a good method of image denoising. In the early days, most models were modeled based on the image prior method. For example, the non-local mean (NLM) algorithm [4] estimated the center point of the reference block using the weighted average of self-similar blocks to reduce noise. In addition, block-matching and 3D-filtering (BM3D) algorithms [5] enhanced sparsity through cooperative transformation to achieve image denoising. In calculating the weighted nuclear norm minimization (WNNM) [6], prior information was used to determine the nuclear norm for image denoising. Indeed, image denoising can be carried out using image-prior-based methods, and a good denoising effect can be achieved. However, these methods all face two problems [7]: (1) the optimization problem in the test stage is very complex, making the denoising process time-consuming; (2) parameters need to be manually adjusted to obtain a better image-denoising effect.

As evident in models such as AlexNet [8], VGG [9], ResNet [10], etc., deep learning has flourished, and convolutional neural networks (CNN) have been widely used in image denoising and have achieved a better denoising effect. For example, residual learning and bath normalization were applied to a deep convolutional neural network for image denoising (DnCNN) [7] to enhance the learning ability of the model. The downsampled subimages and noise level map were used as model inputs, in order to improve the adaptability of the network to different noises (FFDNet) [11]. Furthermore, the residual dense layers were proposed to be incorporated in CNN (RDN) [12,13] to improve the performance of the model for image super-resolution. Batch renormalization was applied to CNN (BRDNet) [14] to enhance the expressiveness of the models. Make full use of the effects from the shallow layer on the deep layer of the network (ADNet) [15]. In fact, although these models do improve the effect of image denoising, there are still some problems: (1) One of the problems is the image data redundancy, which is represented by similar details in some parts of the image. Furthermore, CNN-based models do not make full use of the global similarity information of the image, which limits the expressive ability of the model. (2) In some methods that use lightweight networks, the effect of image denoising needs to be improved, for example, ADNet and BRDNet, and some methods are complex, such as BM3D. (3) Some methods do not adequately capture key information in complex environments, and the denoising effect is not very good in complex noise and real-world images.

In this paper, we propose a new network for image denoising. Its core is the residual dense attention similarity module (RDASM). First, a shallow feature is extracted through the preprocessing module, which only contains two convolution layers. Then, the local features of the image are captured using the residual dense module (RDM); it includes residual learning (LR [10]) and dense layers (DenseNet [16] and RDN [12]), and leaky ReLU (LReLU [17]) is used to avoid the case of zero gradients. In addition, inspired by BM3D and NLM, an attention mechanism is used to mine the global similarity information of the image, and similar details have similar weights. On this basis, an avg-pooling mechanism is used to better smooth noise points and suppress the noise, and dilated convolution (Dilated Conv [18]) is used to expand the receptive field, so as to better obtain similar global features. Furthermore, the deep features of the image are mined by cascading residual dense attention similarity modules (RDASMs). Finally, through global residual learning, the effect is enhanced from shallow layers to deep layers.

The contributions of this paper are as follows:

1. This paper proposes a new image-denoising algorithm framework: the residual dense attention similarity network (RDASNet). Different from the existing CNN denoising models, the core of the proposed model is the residual dense attention similarity module (RDASM), which extracts the local features of the image through CNN and captures the global similarity features of the image through the attention similarity module. Furthermore, this is very effective for dealing with complex noise images. The proposed model achieves a better denoising effect, both qualitatively and quantitatively; thus, it is more suitable for complex noise.
2. Weight is used to represent the similarity of image details. Data redundancy exists in the image, that is, the textural detail of the whole image is similar. The attention similarity module (ASM) can make full use of the global information of the image, and similar details have similar weights. Furthermore, the ASM gives larger weight to key features. Ablation studies have also shown the effectiveness of ASM.
3. In the attention mechanism, dilated convolution is used to enlarge the receptive field in attention so as to extract the global similarity information better. Furthermore, dilated convolution has a smaller number of parameters. Compared with RDN, the number of parameters in our proposed model increased only by 0.07 M, while PSNR increased by 0.10–0.22 dB.
4. For global pooling in the attention mechanism, we found that the color of the image is dimmer after avg-pooling, which makes the noise in the image look less obvious than before; that is to say, avg-pooling is beneficial to smooth and suppress the noise in the image. Moreover, ablation studies have also shown that avg-pooling could further improve model performance for image denoising.

## 2. Related Work

### 2.1. Deep CNNs for Image Denoising

Due to the abovementioned two main defects, image denoising is based on the image prior knowledge modeling. Moreover, a convolutional neural network can automatically extract features, thus reducing computational costs [19,20]. Therefore, deep CNNs are widely used in image denoising.

Zhang et al. [7] first designed deep CNNs for image denoising (DnCNN), and they improved the performance of the model by stacking multiple convolutional layers, residual learning [10], and batch normalization [21]. DnCNN obtained a better effect than traditional BM3D; therefore, it is a successful application of CNN in image denoising. It was noted that DnCNN has a better effect on certain noises, and the effect of blind noise is not ideal. Then, to deal with this problem, Zhang et al. [11] designed a fast and flexible denoising network (FFDNet). They used a trainable noise level map as the model input, and a single model can handle different noise levels. Furthermore, in order to make full use of the abundant features of all the layers to improve model performance, Zhang et al. [12] proposed a very deep residual dense network (RDN) for image super-resolution, and by cascading residual dense blocks, a continuous memory mechanism was formed. Moreover, to reduce computational costs, Tian et al. [14] proposed a batch renormalization denoising network (BRDNet), which used batch renormalization [22] to accelerate the convergence of network training. Furthermore, it is suitable for denoising on low-configuration hardware devices. Using dilated convolution instead of ordinary convolution can also reduce the number of model parameters. For example, Tian et al. [15] designed ADNet, and they used sparse blocks composed of dilated convolution and ordinary convolution to improve performance and efficiency; in addition, an attention mechanism was used to extract hidden information. Motivated by this, and that deep CNNs have shown better performance for image denoising, we used CNNs for image denoising.

### 2.2. Attention Mechanism and Similarity

Extracting key information in complex environments is very important for image denoising. Furthermore, there is redundant information in the image; specifically, there is a global similarity in image details. A better use of image data redundancy can improve the performance of the model in a complex environment.

The attention mechanism used in this study originated in the human brain [23], then it was introduced into natural language processing [24] and applied to computer vision [25]. From a mathematical point of view, the attention mechanism provides a pattern of weights to perform operations on. In a neural network, the attention mechanism enables the use of some network layers to calculate the corresponding weight value of the feature maps and to carry out the attention mechanism of the feature maps. Furthermore, an attention mechanism can be understood as giving more attention to the most meaningful parts (with more weight) [26]. Thus, this is very useful in complex environments to obtain key information about the image, where attention gives more weight. Jaderberg et al. [27] proposed a spatial transformer network (STNet), which focused on the spatial information of the image. Hu et al. [28] proposed a squeeze-and-excitation network (SENet) to study channel dimensions, which can adaptively adjust the key features of image channel dimensions. Furthermore, ADNet [15] uses only one convolution focus channel information to guide the CNN in training the model. In addition, Woo et al. [29] proposed a plug-and-play convolutional block attention module (CBAM) that extracts global image features in the channel spatial dimension, respectively, through max-pooling and avg-pooling. Inspired by these methods, our attention mechanism also includes two dimensions, channel and spatial dimensions.

Classical image denoising, such as classical NLM [4] and BM3D [5], make full use of image similarity information. NLM gives large weight to neighborhoods with similar pixels, and BM3D searches for similar patches of a given patch. In image denoising with deep CNNs, there are few studies focused on the global similarity of images. Motivated by this, we used an attention mechanism to represent the similarity of image details.

## 3. Proposed RDASNet Denoising Method

**Figure 1:**
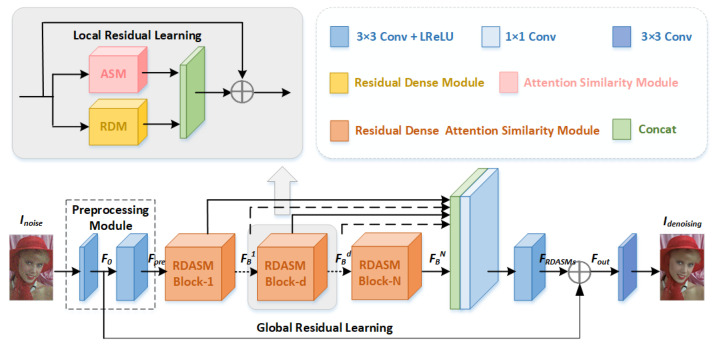
Network architecture of the proposed RDASNet.

In this section, a new image-denoising network is described, along with the residual dense attention similarity network (RDASNet), as shown in Figure 1. Firstly, the shallow information of the image is extracted using the preprocessing module (PM), which only contains two convolution layers. The core of our model is the residual dense attention similarity module (RDASM), which consists of the residual dense module (RDM, motivated by RDN [12]) and the attention similarity module (ASM, motivated by CBAM [29], NLM [4], and BM3D [5]). The RDM captures the local features of the image through residual learning and dense layers, while the ASM uses attention to assign similar weights to areas with similar image details (pixels) and gives large weight to the key features of the image. This is useful for image denoising in a complex background. Then, global residual learning is used to enhance the effect from a shallow layer to a deep layer in the network.

### 3.1. Network Structure

Assume that $I_{noise}$ and $I_{denoising}$ represent the input image containing noise and the output denoising image, as shown in Figure 1. Specifically, in the preprocessing module (PM), we use two convolution and activation layers to extract shallow features with 64 3 × 3 convolution kernels for each layer to obtain the shallow feature map $F_{pre}$ as follows:

(1) $$F_{pre}=H_{pre2}\left(H_{pre1}\left(I_{noise}\right)\right)$$

where $H_{pre1}$ and $H_{pre2}$ denote convolution and activation operations. Furthermore, we use the leaky rectified linear unit (LReLU [17]) activation function.

Then, $F_{pre}$ is sent to the N-stacked residual dense attention similarity module to capture the image features. Through RDASMs, we can obtain $F_{B}^{N}$ using N-RDASM.

(2) $$F_{B}^{N}=H_{B}^{N}\left(F_{B}^{N-1}\right)=H_{B}^{N}\left(\cdots H_{B}^{d}\left(\cdots H_{B}^{1}\left(F_{pre}\right)\cdots \right)\cdots \right)$$

where $H_{B}^{d}$ denotes the operations of the *d*-th RDASM, and $H_{B}^{d}$ is a non-linear transformation; it’s a series of operations, such as convolution and LReLU. More details on RDASM are given in Section 3.2. Then, the output features of all RDASMs are fused, and the output feature $F_{RDASMs}$ can be obtained.

(3) $$F_{RDASMs}=H_{F2}\left(H_{F1}\left(\left[F_{B}^{1},\cdots ,F_{B}^{d},\cdots ,F_{B}^{N}\right]\right)\right)$$

where $\left[F_{B}^{1},\cdots ,F_{B}^{d},\cdots ,F_{B}^{N}\right]$ indicates that the feature maps from 1 to N RDASM will be concatenated. $H_{F1}$ means to control the number of output channels with a 1 × 1 convolution, and $H_{F2}$ means that a 3 × 3 convolution is used to improve the expression ability of the model.

Finally, we use global residual learning to enhance the effect from a shallow layer to a deep layer, and thus we can obtain the output feature map $F_{out}$.

(4) $$F_{out}=F_{RDASMs}+F_{0}$$

Then, through a 3 × 3 convolution, it will be converted to three channels or one channel (depending on whether the input is a color image or a gray image).

(5) $$I_{denoising}=H_{out}\left(F_{out}\right)$$

where $I_{denoising}$ is the final output of our model, that is, the image after denoising through the RDASNet.

In addition, the L1 loss function is used to optimize the difference between the denoising output image $I_{denoising}$ of our model and the ground truth image $I_{GT}$. Assuming that the training set has N pairs of images $I_{noise}^{i}$, $I_{GT}^{i}$ (i=1,2,⋯,N), the loss function of RDASNet can be calculated by

(6) $$L=\frac{1}{N}\sum_{i=1}^{N}\left|F_{RDASNet}\left(I_{noise}^{i}\right)-I_{GT}^{i}\right|$$

where $F_{RDASNet}\left(\cdot \right)$ denotes the predicted output of the model.

### 3.2. Residual Dense Attention Similarity Module (RDASM)

**Figure 2:**
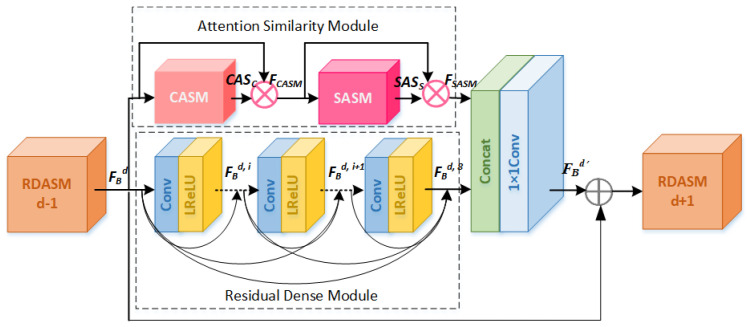
Residual dense attention similarity module. CASM and SASM represent the channel attention similarity module and spatial attention similarity module, respectively.

Our proposed residual dense attention similarity module is the core of RDASM; it includes the residual dense module (RDM) and the attention similarity module (ASM), as shown in Figure 2. RDM is used to obtain the local features of the image, form dense layers through a series convolution, and enhance the representation of the model through residual learning. ASM is used to obtain key similarity features in a complex background, including channel attention similarity (CASM) and spatial attention similarity (SASM). CASM focuses on the global image similarity information from the channel dimension, while SASM focuses on the global image similarity information from the spatial dimension.

In Figure 2, we can obtain the channel attention similarity map $M_{C}\in R^{C\times 1\times 1}$, and we can obtain the spatial attention similarity map $M_{S}\in R^{1\times H\times W}$. Then, the attention similarity module can be described as

(7) $$\begin{array}{c} F_{CASM}=CAS_{C}⊗F_{B}^{d}\\ F_{SASM}=SAS_{S}⊗F_{CASM} \end{array}$$

where $F_{B}^{d}$ denotes the input feature map of the *d*-th RDASM. ⊗ denotes element-wise multiplication. More details on CASM and SASM are given in Section 3.2.2 and Section 3.2.3, respectively.

Then, the output $F_{SASM}$ of the attention similarity module and the serial output $F_{B}^{d,i}$ of the residual dense more are concatenated (more details are provided in Section 3.2.1).

(8) $$F_{B}^{d^{\prime }}=H_{B}^{d}\left(\left[F_{B}^{d,0},\cdots ,F_{B}^{d,i},\cdots ,F_{B}^{d}d,c-1,F_{SASM}\right]\right)$$

where $H_{B}^{d}$ denotes the $\left[F_{B}^{d,0},\cdots ,F_{B}^{d,i},\cdots ,F_{B}^{d}d,c-1,F_{SASM}\right]$ are concatenated. Then, the 1 × 1 convolution is used to control the number of output channels. Thus, the input feature map of *d* + 1-th RDASM can be obtained by

(9) $$F_{B}^{d+1}=F_{B}^{d^{\prime }}+F_{B}^{d}$$

where $F_{B}^{d+1}$ denotes the output of *d* + 1-th RDASM, and the feature map of the current layer is passed backward through local residual learning.

#### 3.2.1. Residual Dense Module (RDM)

The residual dense module is designed with reference to RDN, and each RDM block adopts an eight-layer convolution and activation operation to achieve contiguous memory by transferring the feature $F_{B}^{d}$ of the current layer to each subsequent layer, respectively, so as to make full use of the features of each layer, as shown in Figure 2. The output feature map $F_{B}^{d,c}$ of the *c*-th Conv layer of the *d*-th RDASM can be expressed as

(10) $$F_{B}^{d,c}=\sigma \left(W_{B}^{d,c}\left[F_{B}^{d,0},\cdots ,F_{B}^{d,i},\cdots ,F_{B}^{d,c-1}\right]\right)$$

where $F_{B}^{d}=F_{B}^{d,0}$. $F_{B}^{d,i}$ is the feature map extracted from the *i*-th (*i* = 0, 1, ⋯, *c*−1) Conv layer of the *d*-th RDASM, and $W_{B}^{d,c}$ is the weight of the *c*-th Conv layer. σ denotes the LReLU activation function.

#### 3.2.2. Channel Attention Similarity Module (CASM)

**Figure 3:**
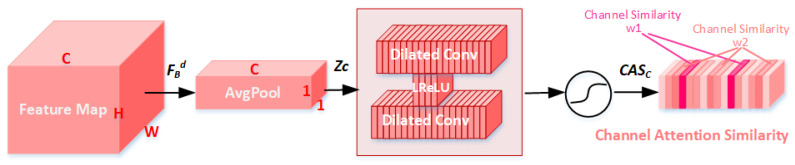
Channel attention similarity module. Channels with similar pixels have similar weights, and key channel features have larger weights. The two dark red channels in the figure are similar and have a similar weight w1.

CASM uses global avg-pooling on each channel to compress the feature map from C × H × W to C × 1 × 1 so that one pixel represents one channel to achieve global information embedding [28], as shown in Figure 3. We use $F_{B}^{d}\in R^{C\times H\times W}$ to represent the feature map input to the CASM. The global spatial information is compressed into a channel descriptor $z\in R^{C\times 1\times 1}$ through global avg-pooling. Furthermore, the *c*-th channel of $z_{c}$ is calculated by

(11) $$z_{c}=H_{GAP}\left(f_{c}\right)=\frac{1}{H\times W}\sum_{i=1}^{H}\sum_{j=1}^{W}f_{c}\left(i,j\right)$$

where $H_{GAP}$ is used to represent global avg-pooling. Compared with max-pooling, avg-pooling is beneficial to smooth and suppress noise and achieve a better denoising effect (more details are provided in Section 4.5). Furthermore, $f_{c}\left(i,j\right)$ represents the value of position (i,j) of feature map $F_{B}^{d}$.

Then, through Dilated+LReLU+Dilated, a gating mechanism with a sigmoid activation is used to learn the interrelationships between the channels. Furthermore, we can obtain the channel attention similarity map $CAS_{c}\in R^{C\times 1\times 1}$. It should be noted that here, we use dilated convolution [18] instead of standard convolution, and the dilation rate is 2. There are two reasons: (1) dilated convolution can enlarge the receptive field, which is beneficial to obtain better global similarity information; (2) there are fewer dilated convolution parameters.

(12) $$CAS_{C}=\sigma_{2}\left(H_{DConv2}\left(\sigma_{1}\left(H_{DConv1}\left(z_{c}\right)\right)\right)\right)$$

where $\sigma_{2}$ denotes the sigmoid activation function. $H_{DConv1}\in R^{\frac{C}{r}\times C}$ and $H_{DConv2}\in R^{C\times \frac{C}{r}}$ represent the two layers of the dilated convolution, respectively. The *r* is the reduction ratio, and then the number of parameters is reduced and set to 16 [28]. $\sigma_{1}$ represents the LReLU activation function after the first dilated convolution layer. Furthermore, $CAS_{c}$ is the channel attention similarity obtained through the CASM, as shown in Figure 3. In addition, similar channel pixels have similar weights. The two channels in dark red in Figure 3 have similar weights w1. Furthermore, CASM gives a large weight to key channel features.

#### 3.2.3. Spatial Attention Similarity Module (SASM)

**Figure 4:**
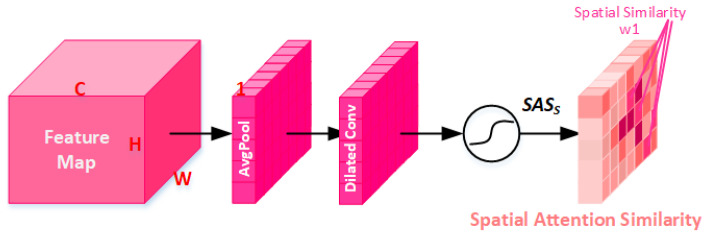
Spatial attention similarity module. Spatial similar regions have similar weights, and spatial key features have larger weights.

SASM also uses global average pooling to compress the channel in the spatial dimension from C × H × W to 1 × H × W, as shown in Figure 4. We use $F_{CAM}\in R^{C\times H\times W}$ to represent the input feature map of the spatial attention module. Then, It is compressed to $R^{1\times H\times W}$ using global average pooling. Then, through a set of non-linear transformation processes, we can obtain the feature map $SAS_{S}\in R^{1\times H\times W}$ of the spatial attention similarity.

(13) $$SAS_{S}=\sigma_{2}\left(H_{DConv}\left(H_{GAP}\left(F_{CASM}\right)\right)\right)$$

where $H_{DConv}$ denotes the dilation convolution, the kernel size is 3 × 3, and the dilation rate is 2. $\sigma_{2}$ denotes the sigmoid activation function.

### 3.3. Implementation Details

In the model, a 3 × 3 convolution kernel was used in all cases without special instructions. Furthermore, we used a zero-padding strategy to keep the size of the image constant. At the same time, a 1 × 1 convolution kernel was used behind each concatenation layer to control the number of output channels. In addition, in the attention similarity module, we used dilated convolution instead of standard convolution, to enlarge the receptive field and reduce the number of parameters. Moreover, RDASMs had a total of 16 residual dense attention similarity modules, and there were 8 levels of convolution in each RDASM.

## 4. Experiment Results

In this section, we present the experimental setup of the model, the experimental results, and the corresponding ablation experiments.

### 4.1. Datasets

#### 4.1.1. Train Datasets

The training datasets consisted of three types: gray image datasets, color image datasets, and real noisy image datasets. The gray image datasets were trained using blind noise and consisted of two public datasets, namely the Waterloo Exploration Database [30] and the BSD400 dataset [11,19]. The BSD400 dataset was randomly selected from ImageNet’s [31] validation set and stored in PNG format. The Waterloo Exploration Database consists of 4744 natural images in PNG format. The color image datasets included the Waterloo Exploration Database and BSD432 [7]. The BSD432 dataset is derived from the Berkeley Segmentation datasets and contains 432 color images. The real noisy image datasets used polyU-Real-World-Noisy-Images datasets [32] to train the model. The polyU-Real-World-Noisy-Images datasets consists of 100 color images with real noise, which are obtained from five cameras: Sony A7 II, Nikon D800, Canon 80D, Canon 600D, and Canon 5D Mark II.

#### 4.1.2. Test Datasets

Similarly, the test datasets also included gray image datasets, color image datasets, and real noisy image datasets. The gray image datasets consisted of Set12 and BSD68 [7]. Set12 has 12 gray images, while BSD68 has 68 gray images. The color image test datasets included CBSD68, McMaster [33], and Kodak24 [34]. McMaster and Kodak24 contain 18 and 24 color images, respectively. The real noisy image test dataset was cc [35]. The cc contains 15 real noisy images from different ISO (1600, 3200, and 6400).

### 4.2. Experimental Settings

The main parameters of model training are shown in Table 1. For gray images, the patch size was set to 80 × 80; for color images, it was set to 80 × 80; and for real noisy images, it was set to 64 × 64. In gray image datasets, color image datasets, and real noisy image datasets, we trained 400, 400, and 65 epochs, respectively. In addition, we set the initial learning rate at $1\times 10^{-4}$, and LR remained unchanged in the first 80% of the epochs and subsequently changed to 0.1 times the original with each epoch. Furthermore, in each epoch, we obtained a blind noisy patch by adding the AWGN of noise range σ=[0,75] to the clean patch.

### 4.3. Quantitative and Qualitative Evaluation

#### 4.3.1. RDASNet for Gray Image Denoising

For gray image denoising, we chose several state-of-art denoising methods with the same test datasets, including BM3D [5], DnCNN [7], FFDNet [11], BRDNet [14], ADNet [15], and RDN [13]. In addition, BM3D is a denoising method based on the prior knowledge of images; DnCNN, BRDNet, and ADNet are image-denoising non-blind methods based on CNNs, and FFDNet is blind image denoising. It should be noted that the design of our residual dense module was inspired by RDN, and the noise levels of RDN test datasets are different from other methods, so we retrained the RDN. Moreover, BM3D, DnCNN, FFDNet, BRDNet, and ADNet yielded PSNR, and with direct reference to this, the SSIM was recalculated.

Table 2 and Table 3 report the PSNR and SSIM results on Set12 and BSD68 datasets, respectively. In terms of the quantitative results, our RDASNet achieved the same or better results in most cases than all other methods. The quantitative results of RDASNet were mostly optimal or suboptimal. In particular, in the complex noise, our model was superior to all the most advanced image-denoising methods, mainly because more global image similarity information is paid attention to using our proposed model.

**Figure 5:**
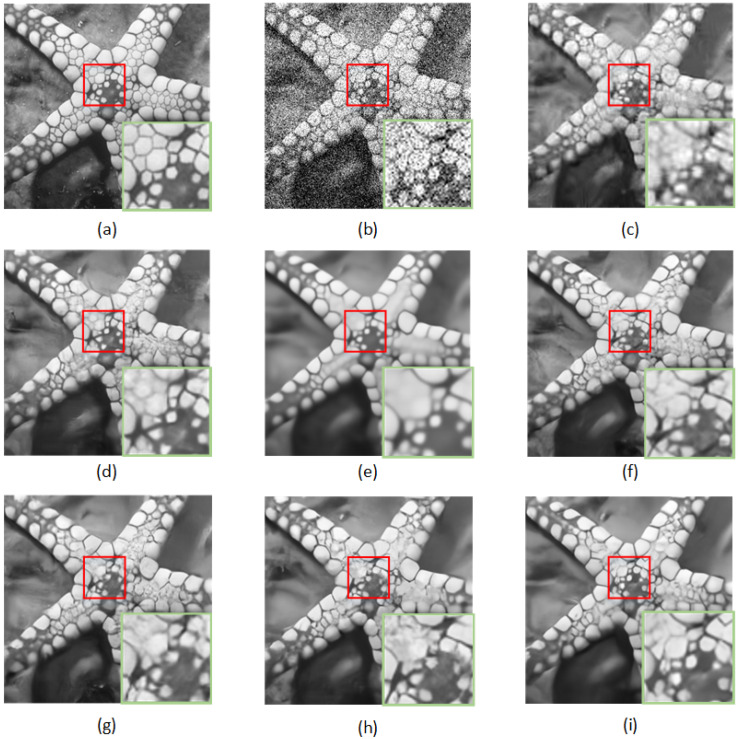
Denoising results of different methods on one image from Set12 with noise level σ=50: (**a**) original image, (**b**) noisy image, (**c**) BM3D/25.04 dB, (**d**) DnCNN/25.70 dB, (**e**) FFDNet/25.75 dB, (**f**) BRDNet/25.77 dB, (**g**) ADNet/25.70 dB, (**h**) RDN/25.65 dB, and (**i**) RDASNet/25.94 dB.

**Figure 6:**
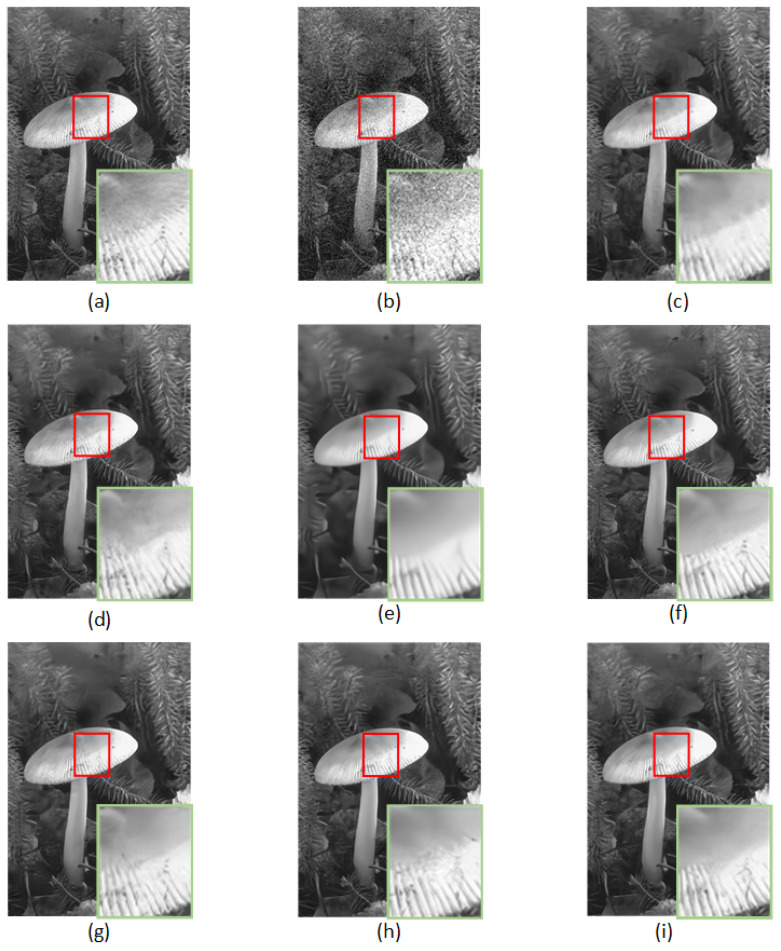
Denoising results of different methods on one image from BSD68 with noise level σ=25: (**a**) original image, (**b**) noisy image, (**c**) BM3D/28.42 dB, (**d**) DnCNN/29.11 dB, (**e**) FFDNet/29.16 dB, (**f**) BRDNet/29.26 dB, (**g**) ADNet/29.11 dB, (**h**) RDN/29.17 dB, and (**i**) RDASNet/29.21 dB.

Figure 5 and Figure 6 show the visualization results. It can be found that the RDASNet proposed by us obtained clearer results and more advanced details. Taking “Starfish.png” in Figure 5 as an example, The effect of BM3D restoration was the least ideal, and other methods, such as FFDNet, BRDNet, ADNet, etc., all had different degrees of distortion. In contrast, our RDASNet could better alleviate blur and restore more image details.

#### 4.3.2. RDASNet for Color Image Denoising

For color image denoising, we compared RDASNet with CBM3D [5], DnCNN, FFDNet, BRDNet, ADNet, and RDN [13].

**Figure 7:**
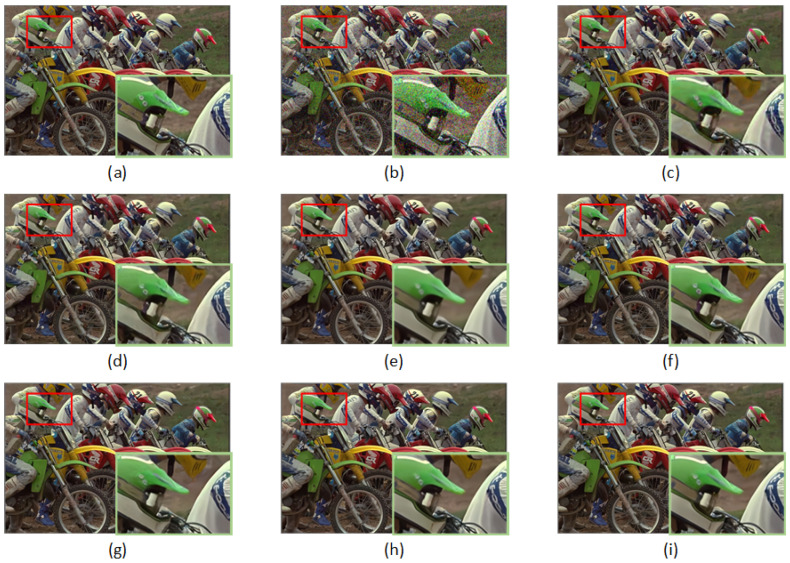
Denoising results of different methods on one image from Kodak24 with noise level σ=35: (**a**) original image, (**b**) noisy image, (**c**) CBM3D/27.33 dB, (**d**) DnCNN/28.43 dB, (**e**) FFDNet/28.60 dB, (**f**) BRDNet/28.88 dB, (**g**) ADNet/28.68 dB, (**h**) RDN/28.94 dB, and (**i**) RDASNet/29.11 dB.

**Figure 8:**
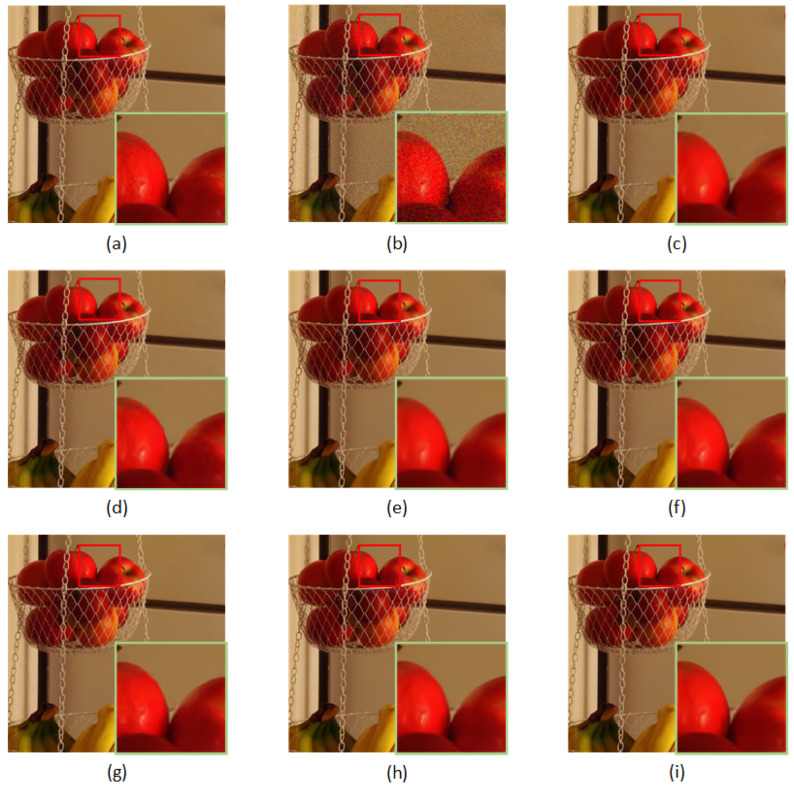
Denoising results of different methods on one image from McMaster with noise level σ=15: (**a**) original image, (**b**) noisy image, (**c**) CBM3D/35.49 dB, (**d**) DnCNN/35.63 dB, (**e**) FFDNet/36.64 dB, (**f**) BRDNet/37.20 dB, (**g**) ADNet/36.59 dB, (**h**) RDN/37.20 dB, and (**i**) RDASNet/37.39 dB.

Table 4 reports the PSNR and SSIM results using the CBSD68, Kodak24, and McMaster datasets. From the quantitative results, it can be inferred that the RDASNet proposed by us outperformed all the other image-denoising methods on color images. This is mainly due to the fact that our model can pay more attention to the global information of the image. Furthermore, Figure 7 and Figure 8 show the visualization results.

#### 4.3.3. RDASNet for Real Noisy Image Denoising

For real noisy images, we chose several commonly used image-denoising methods with the same test datasets, such as CBM3D, WNNM [6], DnCNN, BRDNet, ADNet, and RDN. Table 5 reports the PSNR results using the cc dataset. From Table 5, we can observe that the model proposed by us still achieved the best effect of denoising for real images in terms of the overall mean value. Furthermore, in contrast to other methods, our RDASNet had a good denoising effect taken by different camera devices and could better adapt to different devices.

### 4.4. Ablation Studies

#### 4.4.1. RDASM Design

On the one hand, CNN extracts the image features of the fixed receptive field through convolution operation and pays more attention to the local information of the image. In addition, there are two methods to enlarge the receptive field of convolution [36]: (1) using a larger convolution kernel, for example, a 5 × 5 or 7 × 7 convolution kernel instead of a 3 × 3 convolution kernel; (2) deepening the network layer. Both methods lead to a surge in the number of parameters in the model. On the other hand, there is redundant information in the image; that is, some details of the whole image are similar. Classical image-denoising methods, such as NLM [4] and BM3D [5], obtain better performance by using image similarity. The core idea of the NLM is that the estimate of the current pixel is obtained using the weighted average of the pixels with similar structures in the image. Furthermore, BM3D is a block-matching and 3D-filtering method, and during the block-matching process, similar blocks are found and then filtered. However, in image denoising, few CNN models make full use of the global similarity information of the image, which limits the representation ability of the models. Inspired by this, we designed the RDASM.

RDASM Includes the RDM and ASM. The RDM is formed through residual learning and dense convolution layers, as shown in Figure 2. The RDM focuses on the local information of the image and extracts its features. The ASM consists of CASM and SASM and focuses on the global similarity information of the images from two dimensions of channel and space, respectively, as shown in Figure 3 and Figure 4. The ASM we designed has several differences: (1) It uses an attention mechanism to mine global similarity information. Through CASM or SASM, the channel attention similarity or spatial attention similarity map can be obtained. Similar image details have similar weights, and key features are given larger weights. (2) Dilated convolution is used to enlarge the receptive field, so as to better focus on the global information of the image, and the number of parameters is less than standard convolution. (3) Avg-polling is beneficial to smooth and suppress the noise; more detail can be found in Section 4.4.2.

In order to verify the effectiveness of the RDASM, especially the effectiveness of the attention similarity module (ASM), we designed a set of comparative experiments to compare the merits and demerits of the RDM and RDASM in image denoising on multiple datasets. The results in Table 6 show that the proposed RDASM has a better effect on image denoising, which proves the effectiveness of the proposed structure.

**Figure 9:**
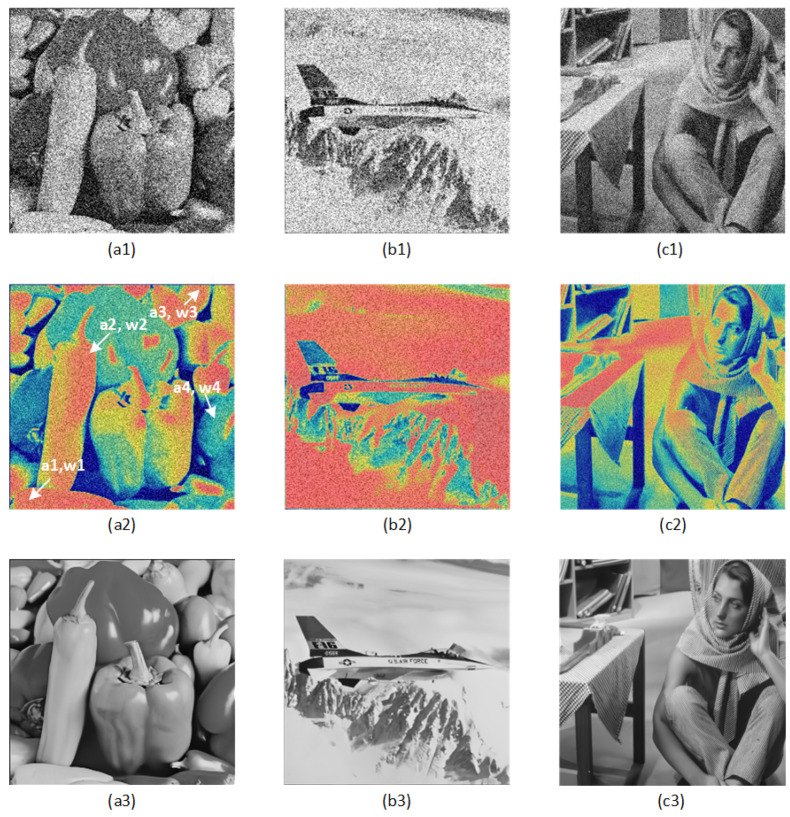
The thermodynamic image of RDASM is proposed. (**a1**–**a3**) are the noise images, (**b1**–**b3**) are the corresponding heatmaps, (**c1**–**c3**) are the corresponding denoising images. In (**a2**) figure, the details of (**a1**–**a3**) are similar, and the weights w1, w2, and w3 are similar. Red indicates high weight, and attention gives more weight to key features.

In addition, we visualized the proposed RDASM, as shown in Figure 9. In Figure 9, label (*1) is the noisy image, (*2) is the heatmap, and (*3) is the denoising image. In (a2) heatmap, we can see that the lower left corner a1, the middle a2, and the upper right corner a3 of the image have similar details and similar weights. Furthermore, in the figure (a3) denoising image, it can be seen that the details of a1, a2, and a3 are indeed very similar. In addition, the attention mechanism gives more weight to the key features (red in the figure indicates large weight), and therefore a2’s weight w2 is larger than a4’s w4. This shows that through RASM, the model can make full use of the redundant information in the image. The global similarity information, in particular, is very useful in images with complex noisy backgrounds.

#### 4.4.2. Global Pooling Design

As mentioned in the section describing the RDCSAM design, it is global pooling that enables the attention mechanism to pay attention to the global information of the image, so global pooling is very important.

**Figure 10:**
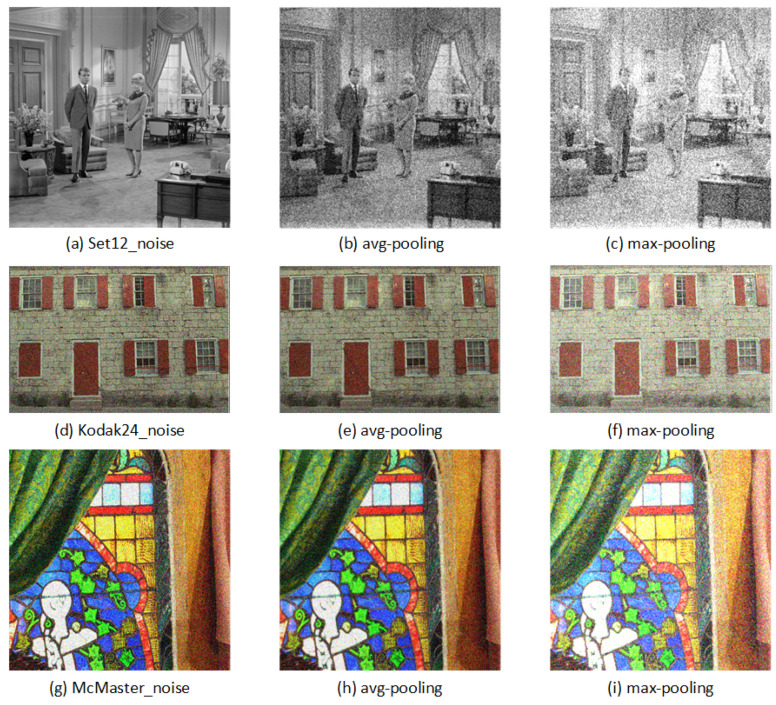
A comparison of avg-pooling and max-pooling. (The noise level σ=50).

Furthermore, here, we made a notable discovery. During an experiment, it was accidentally found that after avg-pooling, the color of an image would be dimmer, and the noise would appear less obvious. However, max-pooling was just the opposite, highlighting the noise, as shown in Figure 10.

In real life, if the light is dim at night, it is difficult to find a small spot on the face, but if it is in a well-lit situation, it is easy to find a small spot on the face. Therefore, we wondered whether the average pooling would have a positive effect on the final performance of the model. Subsequently, experiments were carried out to verify this conjecture.

Then, we only changed the way of global pooling in CBAM and only used average pooling, maximum pooling, and CBAM training models, respectively. In order to save time, the model only trained 200 epochs, and the results are shown in Table 7. This proves once again that we can achieve a better image-denoising effect using avg-pooling; that is, avg-pooling can suppress the noise.

### 4.5. Complexity Analysis

**Figure 11:**
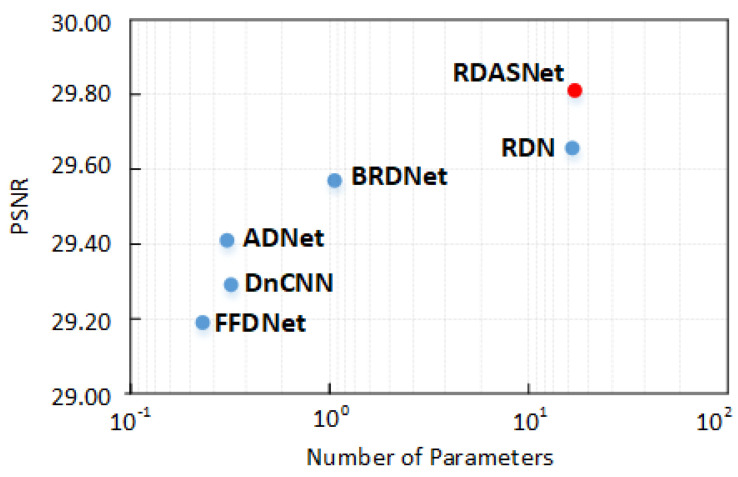
PSNR results on McMaster σ=50 vs. the number of parameters of different methods.

The testing speed of the model is also an important evaluation index. Thus, Table 8 shows the running time results of BM3D, WNNM, DnCNN, FFDNet, BRDNet, RDN, and RDASNet for gray image denoising with sizes of 256 × 256 and 512 × 512, where the noise level is σ=50. Furthermore, we found that compared with RDN, the speed of our model was faster. In addition, we compared the number of parameters with the PSNR of McMaster (σ=50), as shown in Figure 11. ADNet and BRDNet are lightweight models proposed to solve resource-constrained situations, their parameters were smaller, and the performance of our model was better. Our model had high parameters, compared with RDN, and the parameters of our proposed model only increased by 0.07 M, and from Table 2, Table 3 and Table 4, it is evident that PSNR increased by 0.10–0.20 dB for gray images and by 0.11–0.22 dB for color images. The evaluation was conducted in a PyCharm community (2021) environment with an Nvidia GeForce RTX 3090 Ti GPU.

## 5. Conclusions

In this paper, we proposed a residual dense attention similarity network (RDASNet) for image denoising. The local information of the image was extracted using a CNN, and the global information of the image was extracted using the attention similarity module. In addition, our model obtained the shallow features through a preprocessing module; then, the CNN was used to pay more attention to the local information, and the attention similarity module was used to pay more attention to the global similar information, so as to fully benefit from the redundant information of the image. Moreover, similar details had similar weights, and more weight would be given to key features, making the model more suitable for complex noise. Furthermore, global residual learning was used to enhance the effect from a shallow layer to a deep layer. Our proposed RDASNet is more suitable for blind noise, complex environments, and real noise.

In the future, we hope to deploy RDASNet on the mobile end. In general, deployment on mobile platforms requires a smaller model. One possible solution is to use deeply separable convolution instead of traditional convolution to reduce the number of model parameters, or to compress the model through knowledge distillation to design a lightweight RDASNet. 

## Figures and Tables

**Table 1:** Main parameters of RDASNet.

RDASNet	Blind Range	Patch Size	Batch Size	Epochs
gray	[0, 75]	80 × 80	4	400
color	[0, 75]	80 × 80	4	400
real	-	64 × 64	4	65

**Table 2:** PSNR (dB) and SSIM of different methods on the Set12 for different noise levels (i.e., 15, 25, and 50). The best PSNR two results are shown in bold and underlined, respectively.

Images	C.man	House	Peppers	Starfish	Monarch	Airplane	Parrot	Lena	Barbara	Boat	Man	Couple	Average	
					Noise	Level	σ	=	15					
BM3D	31.91	34.93	32.69	31.14	31.85	31.07	31.37	34.26	33.10	32.13	31.92	32.10	32.37/-	
DnCNN	32.61	34.97	33.30	32.20	33.09	31.70	31.83	34.62	32.64	32.42	32.46	32.47	32.86/0.9020	
FFDNet	32.43	35.07	33.25	31.99	32.66	31.57	31.81	34.62	32.54	32.38	32.41	32.46	32.77/0.9055	
BRDNet	32.80	35.27	33.47	32.24	**33.35**	31.85	**32.00**	**34.75**	**32.93**	32.55	**32.50**	32.62	**33.03**/0.9076	
ADNet	**32.81**	35.22	**33.49**	32.17	33.17	**31.86**	31.96	34.71	32.80	**32.57**	32.47	32.58	32.98/0.9031	
RDN	32.33	35.18	33.41	32.14	33.00	31.70	31.87	34.69	32.34	32.42	32.41	32.58	32.84/0.9045	
RDASNet	32.70	**35.28**	33.41	**32.26**	33.18	31.85	31.96	**34.75**	32.76	32.48	32.49	**32.63**	32.99/0.9069	
					Noise	Level	σ	=	25					
BM3D	29.45	32.85	30.16	28.56	29.25	28.42	28.93	32.07	30.71	29.90	29.61	29.71	29.97/-	
DnCNN	30.18	33.06	30.87	29.41	30.28	29.13	29.43	32.44	30.00	30.21	30.10	30.12	30.43/0.8605	
FFDNet	30.10	33.28	30.93	29.32	30.08	29.04	29.44	32.57	30.01	30.25	30.11	30.20	30.44/0.8662	
BRDNet	**31.39**	33.41	31.04	**29.46**	30.50	29.20	29.55	32.65	**30.34**	30.33	30.14	30.28	30.61/0.8669	
ADNet	30.34	33.41	**31.14**	29.41	30.39	29.17	29.49	32.61	30.25	**30.37**	30.08	30.24	30.58/0.8634	
RDN	29.97	33.53	31.11	29.25	30.23	29.14	29.43	32.62	29.72	30.21	30.09	30.31	30.47/0.8644	
RDASNet	30.29	**33.86**	31.12	29.42	**30.51**	**29.21**	**29.61**	**32.80**	30.31	30.35	**30.18**	**30.36**	**30.67**/0.8679	
					Noise	Level	σ	=	50					
BM3D	26.13	29.69	26.68	25.04	25.82	25.10	25.90	29.05	27.22	26.78	26.81	26.46	26.72/-	
DnCNN	27.03	30.00	27.32	25.70	26.78	25.87	26.48	29.39	26.22	27.20	27.24	26.90	27.18/0.7810	
FFDNet	27.05	30.37	27.54	25.75	26.81	25.89	26.57	29.66	26.45	27.33	27.29	27.08	27.32/0.7893	
BRDNet	**27.44**	30.53	27.67	25.77	26.97	25.93	**26.66**	29.73	26.85	27.38	**27.27**	27.17	27.45/0.7915	
ADNet	27.31	30.59	**27.69**	25.70	26.90	25.88	25.56	29.59	26.64	27.35	27.17	27.07	27.37/0.7862	
RDN	27.16	30.77	27.51	25.65	26.93	25.80	26.53	29.75	26.08	27.37	27.25	27.14	27.33/0.7876	
RDASNet	27.34	**31.03**	**27.69**	**25.94**	**26.99**	**26.12**	26.53	**29.80**	**26.89**	**27.41**	27.28	27.29	**27.53**/0.7940	

**Table 3:** PSNR (dB) and SSIM of different methods on the BSD68 for different noise levels (i.e., 15, 25, and 50). The best PSNR two results are shown in bold and underlined, respectively.

Methods	σ=15	σ=25	σ=50
BM3D	31.07/-	28.57/-	25.62/-
DnCNN	31.72/0.8901	29.23/0.8276	26.23/0.7170
FFDNet	31.62/0.8952	29.19/0.8345	26.30/0.7278
BRDNet	**31.79**/0.8966	29.29/0.8346	26.26/0.7284
ADNet	31.74/0.8882	29.25/0.8260	29.29/0.7169
RDN	31.62/0.8943	29.16/0.8314	26.27/0.7223
RDASNet	31.77/0.8960	**29.30**/0.8347	**26.38**/0.7294

**Table 4:** PSNR (dB) and SSIM of different methods on the CBSD68, Kodak24, and McMaster for different noise levels (i.e., 15, 25, 35, 50, and 75). The best PSNR two results are shown in bold and underlined, respectively.

Datasets	Methods	σ=15	σ=25	σ=35	σ=50	σ=75
	CBM3D	33.52/-	30.71/-	28.89/-	27.38/-	25.74/-
	DnCNN	33.98/0.9290	31.31/0.8830	29.65/0.8421	28.01/0.7896	-
	FFDNet	33.80/0.9310	31.18/0.8864	29.58/0.8473	-	26.57/0.7285
CBSD68	BRDNet	34.10/-	31.43/0.8912	29.77/0.8500	28.16/0.8005	26.43/0.7342
	ADNet	33.99/0.9325	31.31/0.8878	29.66/0.8479	28.04/0.7961	26.33/0.7288
	RDN	34.06/0.9342	31.42/0.8907	29.79/0.8522	28.18/0.8016	26.50/0.7376
	RDASNet	**34.17**/0.9354	**31.53**/0.8925	**29.91**/0.8547	**28.31**/0.8055	**26.63**/0.7421
	CBM3D/-	34.28/-	31.68/-	29.90/-	28.46/-	26.82/-
	DnCNN	34.73/0.9209	32.23/0.8775	30.64/0.8398	29.02/0.7917	-
	FFDNet	34.55/0.9234	32.11/0.8818	30.56/0.8455	28.99/0.7993	27.25/0.7373
Kodak24	BRDNet	34.88/-	32.41/0.8869	30.80/0.8485	29.22/0.8029	27.49/0.7442
	ADNet	34.76/0.9250	32.26/0.8830	30.68/0.8461	29.10/0.7990	27.40/0.7637
	RDN	35.02/0.9272	32.55/0.8869	31.00/0.8515	29.45/0.8061	27.80/0.7498
	RDASNet	**35.16**/0.9290	**32.69**/0.8893	**31.16**/0.8548	**29.60**/0.8111	**27.95**/0.7546
	CBM3D	34.06/-	31.66/-	29.92/-	28.51/-	26.79/-
	DnCNN	34.80/-	32.47/-	30.91/-	29.21/-	-
	FFDNet	34.47/0.9224	32.25/0.8894	30.76/0.8599	29.14/0.8201	27.29/0.7635
McMaster	BRDNet	35.08/-	32.75/0.8965	31.15/0.8647	29.52/0.8260	27.72/0.7734
	ADNet	34.93/0.9256	32.56/0.8899	31.00/0.8598	29.36/0.8190	27.53/0.7637
	RDN	35.04/0.9285	32.74/0.8961	31.22/0.8672	29.60/0.8300	27.82/0.7800
	RDASNet	**35.21**/0.9301	**32.91**/0.8988	**31.41**/0.8710	**29.81**/0.8360	**28.04**/0.7884

**Table 5:** PSNR (dB) of different methods using the cc dataset. The best two results are shown in bold and underlined, respectively.

Camera Setting	CBM3D	WNNM	DnCNN	BRDNet	ADNet	RDN	RDASNet
Canon	**39.76**	37.51	37.26	37.63	35.96	35.85	37.05
5D	36.40	33.86	34.13	**37.28**	36.11	36.07	37.14
ISO = 3200	36.37	31.43	34.09	**37.75**	34.49	36.42	36.85
Nikon	34.18	33.46	33.62	34.55	33.94	38.00	**38.09**
D600	35.07	36.09	34.48	35.99	34.33	37.41	**38.14**
ISO = 3200	37.13	**39.86**	35.41	38.62	38.87	35.87	34.47
Nikon	36.81	36.35	35.79	39.22	37.61	39.66	**39.96**
D800	37.76	**39.99**	36.08	39.67	38.24	39.06	39.36
ISO = 1600	37.51	37.15	35.48	**39.04**	36.89	37.91	38.40
Nikon	35.05	**38.60**	34.08	38.28	37.20	37.93	38.33
D800	34.07	36.04	33.70	37.18	35.67	37.73	**37.86**
ISO = 3200	34.42	**39.73**	33.31	38.85	38.09	37.31	38.30
Nikon	31.13	33.29	29.83	32.75	32.24	35.47	**36.25**
D800	31.22	31.16	30.55	**33.24**	32.59	30.61	28.68
ISO = 6400	30.97	31.98	30.09	32.89	33.14	36.49	**37.27**
Average	35.19	35.77	33.86	36.73	35.69	36.79	**37.01**

**Table 6:** PSNR (dB) of RDASM and RDM using the Set12 and BSD68 datasets for different noise levels (i.e., 15, 25, 35, 50, and 75).

Methods	σ=15	σ=25	σ=35	σ=50	σ=75
		Set12			
RDM	32.84	30.47	28.96	27.33	25.54
RDASM	32.99	30.67	29.17	27.53	25.73
		BSD68			
RDM	31.62	29.16	27.70	26.27	24.79
RDASM	31.76	29.30	29.82	26.37	24.90

**Table 7:** PSNR (dB) on Set12, BSD68, CBSD68, Kodak24, and McMaster using different pooling methods.

Methods	Avg-Pooling	Max-Pooling	CBAM
Noise Level		σ=50	
Set12	27.4941	27.4028	27.4138
BSD68	26.3455	26.2740	26.2614
CBSD68	28.3153	28.2431	28.2083
Kodak24	29.5174	29.5124	29.4744
McMaster	29.6908	29.6906	29.6834

**Table 8:** Comparison of parameters and run times.

Methods	Device	PSNR/256 × 256	PSNR/512 × 512	Parameters
BM3D	CPU	25.04/0.59	29.05/2.52	-
WNNM	CPU	25.44/203.1	29.25/773.2	-
DnCNN	GPU	25.70/0.0061	29.39/0.0089	556 K
FFDNet	GPU	25.75/0.0113	29.66/0.0168	490 K
BRDNet	GPU	25.77/0.0631	29.73/0.2018	1115 K
ADNet	GPU	25.70/0.0086	29.59/0.0109	519 K
RDN	GPU	25.65/0.0203	29.75/0.0335	21.97 M
RDASNet	GPU	25.94/0.0194	25.80/0.0201	22.05 M

## Data Availability

Publicly available datasets were analyzed in this study. These data can be found here: [https://ece.uwaterloo.ca/~k29ma/exploration/ (accessed 18 November 2021); https://image-net.org/ (accessed 16 November 2021); https://github.com/csjunxu/PolyU-Real-World-Noisy-Images-Dataset (accessed 19 January 2022); https://ieeexplore.ieee.org/abstract/document/8365806/ (accessed 18 November 2021); http://r0k.us/graphics/kodak (accessed 22 November 2021); http://snam.ml/research/ccnoise/ (accessed 19 January 2022)].
